# Comparative analysis of procedural variations in the social defeat stress paradigm in mice: effects of post-defeat housing conditions and aggressor exposure duration

**DOI:** 10.1186/s13041-026-01325-y

**Published:** 2026-07-07

**Authors:** Hirotaka Shoji, Tsuyoshi Miyakawa

**Affiliations:** Division of Systems Medical Science, Center for Medical Science, Fujita Health University, Toyoake, Aichi 470-1192 Japan

**Keywords:** Psychosocial stress, Behavioral phenotypes, Mouse model, Anxiety, Depression

## Abstract

The chronic social defeat stress (CSDS) paradigm is widely used to model psychosocial stress in rodents. Procedural variations across studies may contribute to diverse behavioral outcomes, underscoring the need to clarify the experimental conditions that influence specific behaviors toward establishing a valid animal model. In this study, we examined the effects of post-defeat housing conditions and aggressor exposure duration, two procedural parameters in the CSDS paradigm, on behavior. Adult male C57BL/6 J mice were subjected to chronic SDS for 10 consecutive days under different conditions varying in (i) post-defeat housing (complete separation from an ICR aggressor vs. cohabitation with the aggressor under sensory contact) and (ii) duration of direct contact with aggressor (short exposure: 5 min vs. long exposure: 10 min). A battery of behavioral tests to assess social interaction and anxiety- and depression-related behaviors was conducted after the CSDS procedures. The proportion of mice classified as susceptible or resilient after CSDS did not differ significantly between the post-defeat housing (separation and cohabitation) groups or between the short- and long-contact duration groups. Regardless of the experimental condition, susceptible mice showed increased body weight, reduced social interaction, and decreases in the distance traveled and time spent in center area in the open field test compared with non-stressed controls, whereas no marked differences were observed in behavioral measures in the forced swim and tail suspension tests. Procedural variations led to differences in behavioral outcomes, with both the cohabitation and long-contact duration groups showing reduced distance traveled and less time spent in the center area in the open field test compared with the separation and short-contact duration groups, respectively. These results demonstrate that procedural variations in the CSDS paradigm shape certain behavioral phenotypes, suggesting that cohabitation and long duration of direct contact with aggressor are effective conditions for producing more pronounced and reproducible behavioral changes. These findings highlight the importance of carefully controlling procedural parameters to obtain reproducible CSDS mouse models and that the stress-induced behavioral changes consistently observed represent characteristic features of this model.

**Supplementary Information:**

The online version contains supplementary material available at 10.1186/s13041-026-01325-y.

## Introduction

Chronic social defeat stress (CSDS), a behavioral paradigm in which a mouse is repeatedly exposed to an aggressive conspecific to induce chronic psychosocial stress, is one of the most widely used approaches for investigating the neurobiological mechanisms underlying stress-related psychiatric disorders, including depression and anxiety [1–3]. Since the introduction of experimental protocols for the CSDS paradigm [4], this procedure has been widely adopted as a standard method for assessing susceptibility and resilience to social stress in preclinical neuroscience studies [2, 3, 5]. A large body of studies using this protocol has reported that socially defeated mice show a wide range of behavioral phenotypes, such as social avoidance, increased anxiety-like and depression-related behaviors, and anhedonia-like behavior [3]. However, findings regarding the induction of depression- and anhedonia-like behaviors by CSDS remain inconsistent across studies, as some studies report increased immobility in the forced swim test and tail suspension test [6–8] and reduced sucrose preference in the two-bottle choice test [2, 7, 9, 10], whereas others find no significant differences in one or more of these behavioral measures [2, 9, 11–15], potentially reflecting subtle variations in experimental procedures and laboratory environments [16]. These findings emphasize the importance to determine reliable and reproducible behavioral changes that characterize the CSDS model to further advance research on stress-related behaviors and their underlying mechanisms using this model.

The standardized CSDS paradigm consists of core procedures, including screening for aggressive mice, repeated social defeat exposure, sensory contact housing, and behavioral assessment using the social interaction test [4]. Social defeat exposure is heterogeneous across studies in terms of the number of defeat days, which ranges from several days to approximately two weeks, and the duration of each session (typically 5–10 min), which may also vary across individuals, even within the same study [4]. Mice are housed in the same cage as the aggressor, separated by a perforated divider, for 24 h between defeat sessions, enabling continuous sensory exposure without physical contact. Such sensory contact housing is generally regarded as a key component of the paradigm, although it may not be essential for inducing stress-related behavioral changes in mice. Some studies have suggested that separation from the aggressor without maintaining close sensory contact results in more pronounced depression-related behaviors [15, 17]. Thus, a systematic comparison of protocols is necessary not only to understand how these factors shape behavior and enable the induction of reliable results, but also as a fundamental step toward developing more valid and robust CSDS models.

Here, the present study aimed to investigate how post-defeat housing conditions (complete separation from the aggressor after SDS sessions versus cohabitation with the aggressor allowing sensory but not physical contact) and the duration of direct contact with the aggressor (short exposure; 5 min versus long exposure; 10 min) in the CSDS paradigm influence physiological and behavioral outcomes, including body weight and behavioral measures in the social interaction, light/dark transition, open field, elevated plus maze, Porsolt forced swim, tail suspension, and sucrose preference tests, for assessing locomotor activity and anxiety-like and depression-related behaviors, in adult male C57BL/6 J mice, an inbred strain widely used in behavioral and neuroscience research, while adhering as closely as possible to the protocol described by Golden et al. (2011) [4].

## Materials and methods

### Animals

Male C57BL/6 J (B6J) mice (JAX Mice stock number 000664) aged 6 weeks were purchased from Jackson Laboratory Japan, Inc. (formerly Charles River Laboratories Japan, Inc.; Kanagawa, Japan). Male Slc:ICR (ICR) retired breeder mice were obtained from Japan SLC, Inc. (Shizuoka, Japan) at six months of age. After arrival at our animal facility, B6J mice were group-housed (four per cage), and ICR mice were singly housed in plastic cages (25.0 × 18.2 × 13.9 cm; CLEA Japan, Inc., Tokyo, Japan) covered with stainless-steel wire lids and filter caps (CLEA Japan, Inc., Tokyo, Japan). The rooms were maintained under a 12-h light/dark cycle (lights on at 7:00 h) at 23 ± 2 °C. All cages were provided with paper chip bedding (Paper Clean; Japan SLC, Inc., Shizuoka, Japan), and all animals were given ad libitum access to food (CRF-1; Oriental Yeast Co., Ltd., Tokyo, Japan) and water throughout the experiment. All experimental procedures were approved by the Institutional Animal Care and Use Committee of the Fujita Health University.

### Social defeat stress procedure

The social defeat stress procedure was conducted according to Golden et al. (2011) [4] with slight modifications.

### Screening for aggressor ICR mice

ICR mice (6–9 months old) were screened over 3–4 consecutive days, and animals with an attack latency of less than 60 s during 180-s sessions toward C57BL/6 J mice (8–14 weeks old, used only for screening) on at least 2 days were selected as aggressors. Aggressors were transferred to the hamster cage (25.7 cm × 48.3 cm × 15.2 cm, PC10196HT; Allentown Inc., NJ, US) with steel-wire tops (WBL1019MMB; Allentown Inc., NJ, US), divided in half by a clear perforated plastic divider, at least 2 days prior to the start of CSDS.

### Chronic social defeat stress

**Fig. 1:**
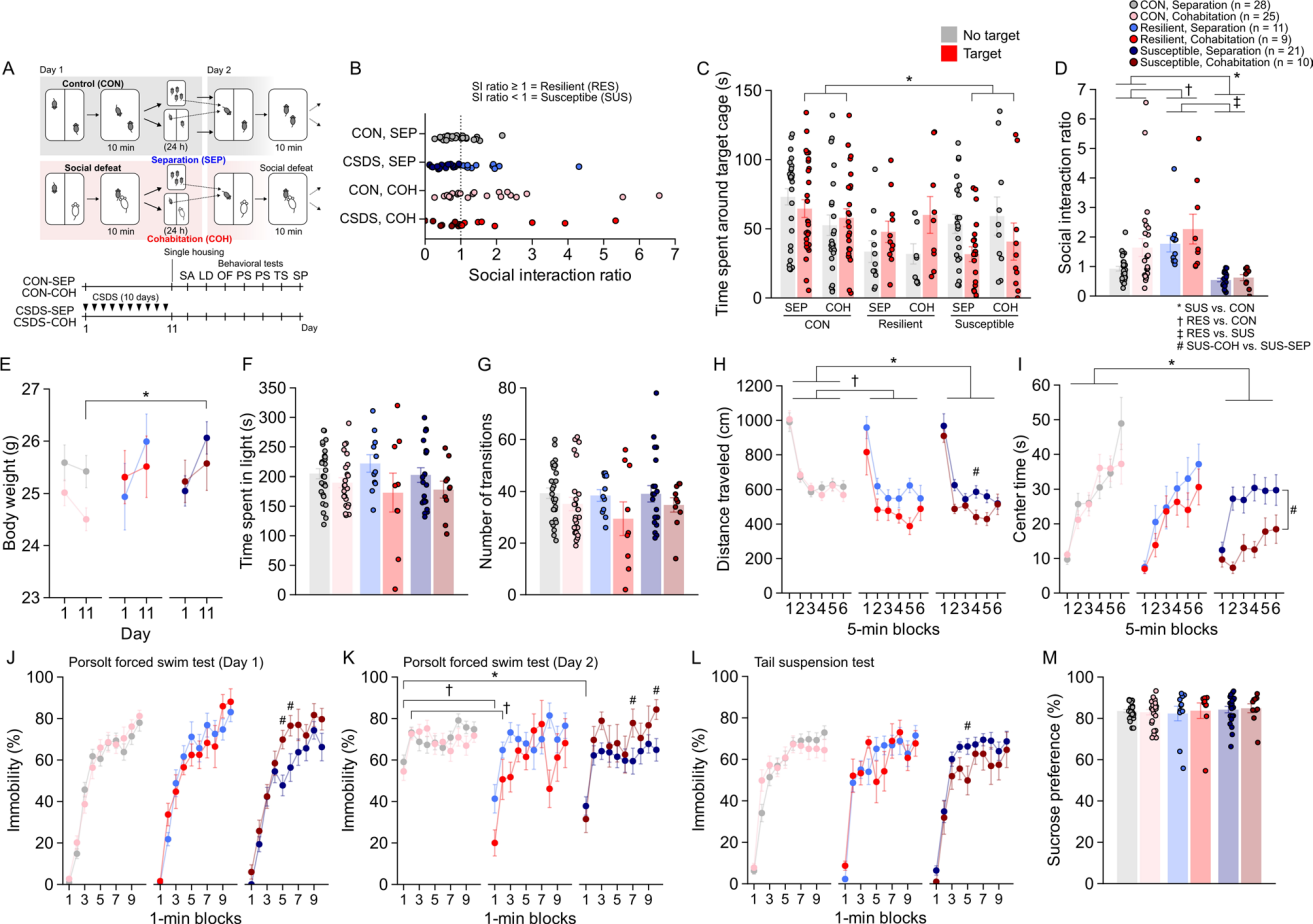
Behavioral outcomes in socially defeated mice under post-defeat separation and cohabitation conditions. **A** Schematic diagram of experimental procedures in Experiment 1. Separation (SEP) housing condition, control and defeated mice were returned to their original group-housing cage after each daily CSDS session; Cohabitation (COH) housing condition, control mice remained in sensory contact with control mice in a hamster cage, while defeated mice remained in sensory contact with aggressor mice between sessions. **B**–**D** Social interaction test: **B** classification of mice into susceptible and resilient groups based on social interaction ratio after CSDS, **C** time spent around target cage (s), and **D** social interaction ratio. **E** Body weight measured before and after CSDS exposure. **F**–**G** Light/dark transition test: **F** time spent in the light chamber (s) and **G** number of transitions between the light and dark chambers. **H**–**I** Open field test: **H** distance traveled (cm) and **I** time spent in center area (s) during 5-min blocks. **J**–**K** Porsolt forced swim test: immobility time (%) for 1 min blocks of the test session on test day 1 (**J**) and on test day 2 (**K**). **L** Immobility time (%) in the tail suspension test. **M** Sucrose preference test: 1% sucrose preference (%). Values are means ± SEM. Statistical analysis was performed by one-, two-, and three-way (repeated measures) ANOVA. *p < 0.05, SUS vs. CON; †*p* < 0.05, RES vs. CON; ‡*p* < 0.05, RES vs. SUS; #*p* < 0.05, SUS-COH vs. SUS-SEP

In Experiment 1 on post-defeat housing conditions, B6J mice were randomly assigned either to a separation (SEP) condition (separation from aggressor/control mice in a hamster cage), in which they were returned to their original group-housing cage after each daily CSDS session, or to a cohabitation (COH) condition, in which they remained in a hamster cage with aggressor/control mice for 24 h while being separated by a perforated divider that allowed sensory contact only (Fig. 1A). Both groups of intruder B6J mice were exposed to social defeat stress for 10 min on 10 consecutive days (days 1–10) by being placed into the compartment of the defeat cage containing a resident ICR aggressor, which was located near the exhaust vent in the animal housing room to minimize odor accumulation. In the SEP group, after the direct contact defeat stress, mice were returned to their home cages (25.0 × 18.2 × 13.9 cm), which were located at least 5 m away from the aggressor cage to minimize potential odor exposure, and group-housed (four per cage) until the next SDS session on the following day, with all mice within each cage assigned to the same housing condition. Mice in the COH group were cohabited for the remainder of the 24-h period in the compartment opposite the aggressor, separated by a perforated divider that permitted sensory contact following defeat sessions. For each daily defeat session, intruder B6J mice were placed in a novel aggressor’s cage. Non-stressed control mice in the SEP group were placed in pairs in the hamster cage, one control animal per side, for the duration of each defeat session and were otherwise group-housed in their home cages. In contrast, the control mice in the COH group were not returned to their home cages but were instead housed on each side of the divider in the cage. In both SEP and COH groups, two control mice from the same condition (SEP or COH) were placed together in a hamster cage. Pairings of the control mice were rotated in a new hamster cage daily and consisted of mice originating either from the same or different home cages. During the 10-day sessions, same-cage pairings occurred on 1–3 sessions, whereas mice from different home cages were paired in the remaining sessions.

**Fig. 2:**
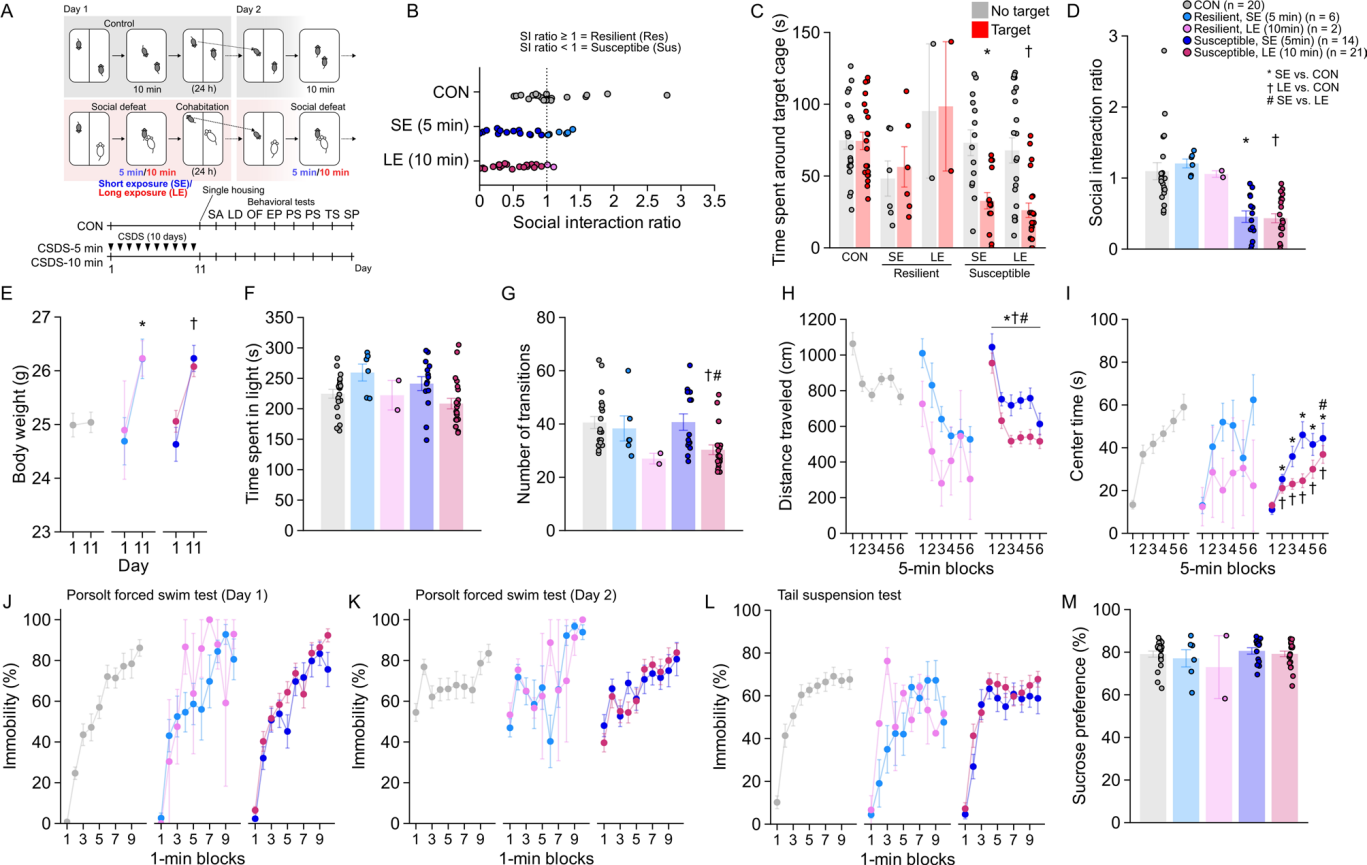
Behavioral outcomes in socially defeated mice under short- and long-exposure conditions of the SDS session. **A** Schematic diagram of experimental procedures in Experiment 2. **B**–**D** Social interaction test: **B** classification of mice into susceptible and resilient groups based on social interaction ratio after CSDS, **C** time spent around target cage (s), and **D** social interaction ratio. **E** Body weight measured before and after CSDS exposure. **F**–**G** Light/dark transition test: **F** time spent in the light chamber (s) and **G** number of transitions between the light and dark chambers. **H**–**I** Open field test: **H** distance traveled (cm) and **I** time spent in center area (s) during 5-min blocks. **J**–**K** Porsolt forced swim test: immobility time (%) for 1 min blocks of the test session on test day 1 (**J**) and on test day 2 (**K**). **L** Immobility time (%) in the tail suspension test. **M** Sucrose preference test: 1% sucrose preference (%). Values are means ± SEM. Statistical analysis was performed by one-, two-, and three-way (repeated measures) ANOVA. *p < 0.05, SE vs. CON; †*p* < 0.05, LE vs. CON; #*p* < 0.05, SE vs. LE

In Experiment 2 on contact duration conditions, mice were individually housed one week prior to the start of the SDS procedure and divided into short- and long-exposure groups (SE and LE groups; Fig. 2A). The SE and LE groups were exposed to the aggressor for 5 and 10 min, respectively, and housed on the opposite side of the aggressor’s compartment for approximately 24 h after direct physical contact with the aggressors. Non-stressed control mice of both groups were housed in pairs on each side of the hamster cage for 24 h and were rotated to a new cage daily. As in Experiment 1, pairings of control mice consisted of mice from either the same or different home cages, with same-cage pairings occurring on 1–3 of the 10 sessions.

Immediately following the last defeat session, all B6J mice in both experiments were singly housed in standard cages on day 11 (after the post-defeat sensory contact session) and were subjected to the social interaction test on day 12. Beginning on day 13, behavioral tests were conducted sequentially in the order shown in Fig. 1A and 2A. Mice that died or were severely injured during CSDS were excluded from the study (Table S1).

### Behavioral tests

#### Social interaction test in the CSDS paradigm (CSDS-SI)

The social interaction test was performed as previously described [4]. The test consisted of two consecutive 150-s sessions in a 40 × 40 × 30 cm gray plastic open-field arena, with illumination set at 100 lx in the center, containing a wire mesh enclosure (10 × 6.5 × 30 cm). In the first session, the enclosure was empty. In the second session, an unfamiliar ICR target mouse was placed inside the enclosure. Mouse movement was recorded using a video camera and analyzed automatically using ImageEP software (see ‘Image analysis’). The time spent in the interaction zone encompasses a 14 cm × 24 cm rectangular area projecting 8 cm around the wire-mesh enclosure and distance traveled (cm) were measured, and a social interaction ratio was calculated by dividing the time spent in the interaction zone when the target is present by the time spent in the interaction zone when the target is absent. Mice with a SI ratio < 1 were defined as susceptible (SUS), whereas those with a ratio ≥ 1 were considered resilient (RES), and all subsequent analyses were conducted based on this classification. Animals for which the SI ratio could not be calculated because the interaction time in the target-absent session was zero were excluded from subsequent analyses (Table S1).

#### Light/dark transition test (LD)

The light/dark transition test was performed as previously described [18, 19]. The apparatus consisted of a cage (21 × 42 × 25 cm) divided into two equal chambers by a partition with a door (O’Hara & Co., Tokyo, Japan). One chamber had white plastic walls and was brightly lit (390 lx) with lights mounted above the ceiling of the chamber. The other chamber had black plastic walls and was dark (2 lx). Both chambers had a light gray plastic floor. The mice were placed in the dark chamber and allowed to move freely between the two chambers for 10 min with the door open. Behavior was recorded using video cameras mounted on the ceiling. The distance traveled (cm), time spent in the light chamber (s), number of transitions between the light and dark chambers, and latency to first enter the light chamber (s) were automatically calculated using video recordings obtained from a camera and ImageLD software.

#### Open field test (OF)

The open field test was conducted in the open field apparatus with the VersaMax activity monitoring system equipped with infrared photobeam sensors (Accuscan Instruments, Columbus, OH, US) to assess locomotor activity and anxiety-like behavior. The open field arena was made of acrylic with transparent walls and a white floor (40 × 40 × 30 cm, inside dimensions). The floor of the center area, defined as 20 × 20 cm, was illuminated at 100 lx. Each mouse was placed in a corner of an open field and allowed to explore freely for 120 min. The distance traveled (cm) was calculated from successive changes in the animal’s position detected by infrared photobeam sensors, and vertical activity was quantified as the number of vertical beam interruptions associated with rearing behavior. Time spent in the center area (s) was measured as the duration during which the animal remained within the predefined central zone of the arena. Stereotypic counts were recorded as repetitive beam interruptions occurring within a restricted area, which may reflect repetitive movements such as head bobbing, sniffing, and grooming. All behavioral measures were collected in 5-min bins over a 120-min test session.

#### Elevated plus maze test (EP)

The elevated plus maze test was conducted as previously described [20]. The apparatus consisted of two open arms (25 × 5 cm) and two closed arms of the same size with 15-cm-high transparent walls (O’Hara & Co., Tokyo, Japan), with illumination set at 100 lx in the central area. The floor of the apparatus was made of light gray plastic and elevated 55 cm above the floor. Each mouse was placed in the central square of the maze facing one of the closed arms. The distance traveled (cm), number of arm entries, percentage of entries into open arms, and percentage of time spent in open arms during a 10-min test period were measured and automatically analyzed using the ImageEP program based on images from a video camera.

#### Porsolt forced swim test (PS)

Mice were placed in a clear plastic cylinder (20 cm height × 10 cm diameter, O’Hara & Co., Tokyo, Japan) filled with water (approximately 21 °C) to a depth of 8 cm for 10 min per day for two consecutive days. The percentage of immobility time was automatically recorded using the ImagePS/TS program based on images acquired from a video camera, as previously described [18, 21].

#### Tail suspension test (TS)

Mice were suspended 30 cm above the floor in a visually isolated area, using adhesive tape placed approximately 1 cm from the tip of the tail. Immobility time was recorded for 10 min using the ImagePS/TS program in the same manner as for the forced swim test.

#### Sucrose preference test (SP)

Mice were given one bottle of water and a second bottle of 1% sucrose solution in their home cages after the tail suspension test. The bottles were weighed prior to testing and again approximately 48 h after the start of the test. Sucrose preference was expressed as 100 × [(sucrose intake)/(sucrose intake + water intake)]. In Experiment 2, food intake per day (g) was calculated as the amount of food consumed over 2 days during the sucrose preference test.

#### Image analysis

Image analysis software (ImageLD/EP/PS/TS) was used to automatically analyze mouse behavior, as previously described [22–24]. The software, based on the public domain ImageJ program (developed by Wayne Rasband at the National Institute of Mental Health, Bethesda), was developed and modified for each test by Tsuyoshi Miyakawa. The ImageLD/EP programs can be freely downloaded from the “Mouse Phenotype Database” (http://www.mouse-phenotype.org/).

#### Statistical analysis

Statistical analyses were performed using SAS Studio (SAS OnDemand for Academics; SAS Institute, Cary, NC, USA) and R (ver. 4.4.2). Behavioral data were analyzed using one-, two- and three-way (repeated measures) ANOVAs with defeat stress conditions and classification (resilient/susceptible) as a between-subject variable and time as a within-subject variable. When an interaction was significant, simple main effect analyses were conducted as appropriate. Group comparisons were performed using the least squares means in SAS PROC GLM and PROC MIXED procedures. Categorical data, resilient/susceptible classification based on SI ratios, were assessed by Fisher’s exact test in R. Statistical significance level was set at 0.05. Data in graphs are presented as mean ± SEM. Effect sizes for each behavioral measure are presented as Hedges’ g with 95% confidence intervals (CIs).

## Results

### Experiment 1: effects of post-defeat housing conditions

The statistical results of the two- or three-way (repeated-measures) ANOVAs conducted for body weight and each behavioral measure are provided in Supplementary Table S2. To examine the effect of post-defeat housing conditions (separation housing condition, in which mice were separated from the hamster cage containing the aggressor/control mouse and returned to their group-housing home cage after each daily CSDS session; SEP, and cohabitation housing condition, in which they remained in a hamster cage with aggressor/control mice for 24 h between sessions; COH) on susceptibility to CSDS, we performed a priori planned comparisons between the two housing condition groups of susceptible mice regardless of whether the stress × housing condition/stress × housing condition × time interaction in the ANOVA was significant. Following the CSDS sessions, 21 out of 32 mice (65.6%) in SEP condition and 10 out of 19 mice (52.6%) in COH condition were classified as susceptible in the social interaction test. The proportions of susceptible (SUS) and resilient (RES) mice did not differ between the post-defeat housing conditions (Fig. 1B: Fisher’s exact test, *p* = 0.3892; odds ratio = 1.70, 95% CI = 0.46–6.36). Three-way repeated-measures ANOVAs indicated a significant main effect of stress and/or a significant stress × session interaction on the time spent in the target area and social interaction ratio (Fig. 1C and 1D). Post-hoc analyses revealed that susceptible mice showed significantly reduced time spent in the interaction zone during the target session (Fig. 1C) and decreased social interaction ratio compared with control mice (Fig. 1D). Resilient mice exhibited significantly higher social interaction ratio than control and susceptible mice (Fig. 1D).

Three-way repeated-measures ANOVA revealed a significant stress × time interaction on body weight, and susceptible mice exhibited significantly higher body weights than control mice following the 10-day CSDS procedure (Fig. 1E). The body weight of resilient mice was intermediate between that of control and susceptible mice and did not differ significantly from that of either the control or susceptible mice (Fig. 1E).

In the light/dark transition test, although there was a significant effect of stress on the distance traveled in the dark chamber, with resilient mice showing a shorter distance traveled in the dark chamber than control mice (Fig. S1A), no significant main effects of stress were observed on the time spent in the light chamber (Fig. 1F), number of transitions between the light and dark chambers (Fig. 1G), distance traveled in the light chamber (Fig. S1B), and latency to first enter the light chamber (Fig. S1C). ANOVAs revealed a significant main effect of housing conditions on the time in light, the number of transitions, and the latency to light chamber, and post-hoc analyses indicated that COH group showed a shorter time spent in the light chamber (Fig. 1F), fewer transitions between the light and dark chambers (Fig. 1G), longer latency to enter the light chamber (Fig. S1C) compared with the SEP group.

Three-way repeated-measures ANOVAs revealed significant main effects of stress on distance traveled, center time, and stereotypic counts in the open field test. Post-hoc analyses indicated that susceptible mice showed significantly decreases in distance traveled (Fig. 1H), center time (Fig. 1I), and stereotypic counts (Fig. S1D) compared with control mice in the open field test, whereas vertical activity was not significantly different between the groups (Fig. S1E). Resilient mice exhibited reductions in distance traveled and stereotypic counts in the open field test than control mice (Fig. 1H and S1D). Post-hoc analyses also indicated that COH group showed reductions in distance traveled, time spent in the center area, and stereotypic counts compared with SEP group (Fig. 1H, I, S1D). Planned comparisons between the SEP and COH groups of susceptible mice revealed that the SUS-COH group displayed reduced distance traveled, center time, and stereotypic counts (Fig. 1H, I, S1D).

In the forced swim test, no significant main effects or interactions were found for immobility on day 1 (Fig. 1J), whereas significant condition × stress and stress × time interactions were observed for immobility on day 2 (Fig. 1K). For distance traveled in the forced swim test, significant main effect of stress and/or significant stress × time/condition × stress × time interactions were detected on days 1 and 2 (Fig. S1F and G). Post-hoc analyses revealed that susceptible mice showed significantly decreased immobility during the first minute of the testing on day 2 (Fig. 1K) and increased distance traveled during the first minute on days 1 and 2 (Fig. S1F and G) compared with control mice. Resilient mice exhibited decreased immobility during the first and second minutes on day 2 (Fig. 1K), along with increased distance traveled during the first minute on day 1 and the first and second minutes on day 2 (Fig. S1F and G) compared with control mice. Planned comparisons revealed that the SUS-COH group showed greater immobility (Fig. 1J and K: 5 and 6 min on day 1; 7 and 10 min on day 2, *p* < 0.05) and lower distance traveled (Fig. 1K: 2, 5, and 6 min on day 1, *p* < 0.05) compared with the SUS-SEP group; in contrast, the SUS-COH group showed greater distance traveled at 1 min on day 2 (Fig. S1G: *p* < 0.05). For the tail suspension test, no significant main effects or interactions were found for immobility (Fig. 1L), and planned comparisons showed that the SUS-COH group showed less immobility at 5 min than the SUS-SEP group (Fig. 1L: *p* < 0.05).

No significant effects of stress were found on sucrose preference or water intake in the sucrose preference test (Fig. 1M and S1H), whereas a significant effect of stress was observed on sucrose intake (Fig. S1H). Post-hoc analyses revealed that susceptible and resilient mice showed significantly increased intake of sucrose solution compared with control mice (Fig. S1H). There was also a significant main effect of housing condition on sucrose intake; COH group showed increased sucrose intake compared with SEP group (Fig. S1H).

Taken together, these results indicate that susceptible mice show reduced social interaction, decreased locomotor activity, and increased anxiety-like behavior following CSDS. Resilient mice also exhibited certain behavioral characteristics that partially resembled those of susceptible mice, including reduced locomotor activity in the open field and reduced immobility accompanied by increased distance traveled at the initial time point of the day-2 retest in the forced swim test, relative to control mice.

### Experiment 2: effects of contact duration conditions

The statistical results of the two- or three-way (repeated-measures) ANOVAs conducted for body weight and each behavioral measure are summarized in Supplementary Table S3. Fourteen out of 20 mice (70.0%) in the short-exposure (SE) condition and 21 of 24 mice (87.5%) in the long-exposure (LE) condition were classified as susceptible; this difference was not statistically significant (Fig. 2B: Fisher’s exact test, *p* = 0.1180; odds ratio = 0.23, 95% CI = 0.02–1.53). Resilient mice from both the LE and SE groups were excluded from subsequent analyses due to insufficient numbers of animals (LE: n = 2; SE: n = 6), although the data are shown in Fig. 2. ANOVAs showed a significant main effect of contact duration condition and/or a significant contact duration condition × session interaction on the time spent in the target area and social interaction ratio (Fig. 2C and D). Post-hoc analyses revealed that both SE and LE groups showed significantly decreased social interaction ratio and reduced time spent in the interaction zone during the target session compared with control mice (Fig. 2C and D).

Two-way repeated-measures ANOVA showed a significant stress × time interaction on body weight. Both SE and LE groups had significantly increased body weights after the CSDS compared with control mice (Fig. 2E).

In the light/dark transition test, while there were no significant effects of contact duration condition on the time spent in the light chamber or the latency to enter the light chamber (Fig. 2F and S2C), significant effects of contact duration were found on the number of transitions (Fig. 2G) and distance traveled in the dark and light chambers (Fig. S2A and B). Post-hoc analyses revealed that LE group exhibited significantly fewer transitions and traveled shorter distances in the light and dark chambers than the control and SE groups (Fig. 2G, S2A–C). In the elevated plus maze test, LE group exhibited significantly reduced distance traveled and fewer number of arm entries compared with control mice (Fig. S2F and G), while there were no significant differences between groups in the percentages of open arm entries and the percentage of time spent on open arms (Fig. S2H and I).

Two-way repeated-measures ANOVAs revealed that there were significant main effects of contact duration condition on the distance traveled, center time, and stereotypic counts in the open field test (Fig. 2H, I, S2D), whereas no significant effect of contact duration condition was observed in the vertical activity (Fig. S2E). Both SE and LE groups exhibited reductions in distance traveled, center time, and stereotypic counts compared with control mice (Fig. 2H, I, S2D). LE group traveled significantly shorter distance, spent less time in center area, and had fewer stereotypic counts than SE group.

For the forced swim test, although there were no significant main effects of contact duration condition or no significant interactions on immobility and distance traveled on days 1 and 2, as shown in the comparison between the COH-SUS and control groups in Experiment 1, LE group displayed decreased immobility at the first minute of day 2 compared with control mice (*p* = 0.0466). In the tail suspension test, there were no significant main effect of contact duration condition and no significant interaction on immobility (Fig. 2L).

Analysis of the sucrose preference test revealed that LE group exhibited a tendency toward increased sucrose intake compared with the control group (Fig. S2L: main effect of condition, *p* = 0.0599; LE vs. CON, *p* = 0.0201), whereas no significant effect of contact duration condition on sucrose preference was found (Fig. 2M). During the test, the LE group consumed significantly more food than the control group (Fig. S2M).

Overall, these data indicate that mice subjected to the 10-min contact duration condition exhibited reduced locomotor activity and increased anxiety-like behavior compared with those subjected to the 5-min condition, suggesting a more pronounced stress response.

### Effect size–based comparison of CSDS-induced behavioral changes

**Fig. 3:**
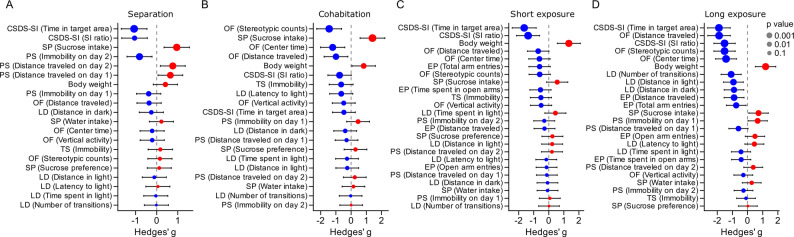
Effect size–based comparison of CSDS-induced behavioral changes. **A**–**B** Comparison of CSDS-induced behavioral changes across behavioral measures using Hedges’ g effect sizes calculated from susceptible vs. control mice under separation housing condition (**A**) and cohabitation housing condition (**B**) in Experiment 1. **C**–**D** Hedges’ g–based ranking of behavioral measures in susceptible vs. control mice in short exposure condition (**C**) and long exposure condition (**D**) in Experiment 2. Red circles represent positive effect sizes (increased in susceptible mice), and blue circles represent negative effect sizes (decreased in susceptible mice)

To better understand the behavioral measures sensitive to CSDS, Hedges’ g effect sizes were calculated for each measure by comparing susceptible and control mice under each experimental condition (Fig. 3). In the SEP and SE conditions, social interaction time/ratio exhibited relatively large effect sizes, whereas effect sizes for open-field measures were generally modest (Fig. 3A and C). In contrast, the COH and long-exposure (LE) conditions showed larger and more consistent effects across multiple open-field parameters. As shown in Fig. 3B and D, increased body weight, reduced social interaction time/ratio, decreases in distance traveled, center time, and stereotypic counts in the open field test, and increased sucrose intake, but not sucrose preference, were ranked among the highest in effect size across the conditions. These findings suggest that social avoidance and open-field behavioral alterations are among the most robust behavioral features associated with stress susceptibility in the CSDS model.

## Discussion

The present study investigated the effects of post-defeat housing conditions and aggressor exposure duration on behavior in the CSDS paradigm in adult male C57BL/6J mice to clarify essential procedural parameters for establishing a valid CSDS model and to understand the core behavioral features of this model. The present study, conducted in accordance with the standardized protocol introduced by Golden et al. (2011) [4], demonstrated that 10-day CSDS exposure induced specific physiological and behavioral outcomes, including increased body weight gain, reduced social interaction, decreased locomotor activity, and reduced time spent in center area in the open field. In contrast, increased immobility in the forced swim and tail suspension tests and decreased sucrose preference, which are generally considered indices of depression-like and anhedonia-like states, were not observed in defeated mice. The two independent experiments in our study yielded similar behavioral results, supporting the reproducibility of the observed effects and suggesting that some behavioral domains may be less sensitive to CSDS, at least under the experimental conditions used in this study. These findings indicate that the CSDS-induced behavioral changes represent robust and reproducible phenotypes reflecting the core features of the SDS model.

This study found that cohabitation with the aggressor after SDS sessions induced more pronounced behavioral alterations than separation from the aggressor cage, as indicated by reduced distance traveled and center time in the open field test and increased immobility and reduced distance traveled in the forced swim test, in susceptible mice. These results indicate that prolonged sensory contact with an aggressor plays a critical role in exacerbating stress-related behavioral responses. In addition, longer exposure to SDS involving direct physical interaction with the aggressor resulted in decreased distance traveled and reduced center time in the open field test compared with shorter exposure. These behavioral alterations, consistent with expectations from the CSDS paradigm, demonstrate that these procedural parameters are essential for inducing decreased locomotor activity and increased anxiety-like behavior and for establishing a reproducible CSDS mouse model. Nevertheless, our findings and those of other studies [15, 25–27] indicate that CSDS even under separation housing and short exposure conditions can induce similar behavioral alterations, which has important ethical implications for minimizing animal burden by adopting those experimental procedures.

Social defeat stress has been reported to induce an increase or decrease in body weight across studies [2, 10, 12, 28]. In this study, defeated mice showed increased body weight after CSDS, consistent with some previous studies [11, 12, 28, 29]. Although the underlying mechanisms of body weight changes remain largely unclear, our results suggest that the increase in body weight may be partly result from stress-induced hyperphagia, which has been reported in human patients with depression [30], as supported by the increased food and sucrose intake observed in defeated mice in the present study. The results of depression-related behavioral tests, such as the forced swim test, tail suspension test, and sucrose preference test following CSDS also vary considerably across studies [2, 6–10, 12–15]. In line with some prior studies [2, 11–13, 15], no marked behavioral differences in immobility in the forced swim and tail suspension tests between defeated and control mice were observed in the present study. These findings suggest that these behavioral measures may not serve as robust indicators of the CSDS model and are highly dependent on experimental conditions, warranting further investigation into the effects of different experimental conditions. It has been suggested that the immobility observed in the forced swim test may reflect a learned and memory-dependent coping strategy [31], whereby animals acquire and recall that escape-directed behaviors are ineffective. The decrease in immobility and/or increase in distance traveled during the early phase of the retest, particularly in the COH and LE groups of defeated mice, may reflect an attenuation of such a learned response, possibly indicating impaired retention or recall of the prior experience with forced swim stress. However, this interpretation should be viewed with caution, as learning and memory functions were not directly assessed in the present study. Future studies will be required to determine whether the reduced immobility observed at the specific time point following CSDS is associated with impaired memory functions.

In this study, a relatively high proportion of control mice exhibited a lower social interaction ratio. Although the exact reasons for these results remain unclear, one possible explanation is that the experimental procedure, in which mice were transferred to different cages and repeatedly exposed to different conspecifics, many of which were unfamiliar individuals, throughout the 10-day CSDS sessions, may have influenced their preference for social novelty, resulting in reduced social approach behavior toward a novel ICR mouse. Another possible explanation is the lighting condition used during the social interaction test, because relatively bright illumination is known to suppress exploratory and social approach behaviors in mice [32, 33], potentially contributing to the reduced social interaction observed in some control mice. In addition, the repeated experimental manipulations may have imposed a mild stress burden, which could also have contributed to the modest decrease in body weight observed within the control group.

In conclusion, the present study demonstrates that procedural variations in the CSDS paradigm shape behavioral outcomes, particularly locomotor activity and anxiety-like behavior in the open field, in addition to social avoidance behavior, a core depression-related phenotype induced by CSDS. These findings highlight the importance of carefully controlling experimental parameters, including post-defeat housing conditions and aggressor exposure duration, to improve reproducibility and facilitate comparisons across studies using the CSDS mouse model. Although previous studies have reported that additional depression-related phenotypes, such as increased immobility in the forced swim and tail suspension tests and reduced sucrose preference, these outcomes were not consistently observed under the present experimental conditions. Furthermore, reward- and motivation-related behaviors, learning and memory, and the molecular and physiological correlates of the observed behavioral alterations were not examined. Considering the inconsistencies and limitations, future studies examining stress-related biomarkers, inflammatory mediators, and neurobiological measures will be important for elucidating the mechanisms underlying the effects of post-defeat housing conditions and aggressor exposure duration and for strengthening the biological interpretation of the behavioral outcomes found in the present study. Despite these limitations, the present findings revealed robust and reproducible hallmark behavioral characteristics induced by 10-day CSDS in adult male C57BL/6 J mice.

## Supplementary Information

Supplementary Material 1: Supplementary Figure 1 Behavioral outcomes in socially defeated mice under post-defeat separation and cohabitation conditions. (A–C) Light/dark transition test: (A, B) distance traveled in the dark and light chambers (cm) and (C) latency to enter the light chamber (s). (D–E) Open field test: (D) stereotypic counts and (E) vertical activity. (F–G) Porsolt forced swim test: distance traveled (cm) for 1 min blocks of the test session on test day 1 (F) and on test day 2 (G). (H) Sucrose preference test: water intake (g) and 1% sucrose intake (g). Values are means ± SEM. Statistical analysis was performed by one-, two-, and three-way (repeated measures) ANOVA. * p < 0.05, SUS vs. CON; † p < 0.05, RES vs. CON

Supplementary Material 2: Supplementary Figure 2 Behavioral outcomes in socially defeated mice under short- and long-exposure conditions of the SDS session. (A–C) Light/dark transition test: (A, B) distance traveled in the dark and light chambers (cm) and (C) latency to enter the light chamber (s). (D–E) Open field test: (D) stereotypic counts and (E) vertical activity. (F–I) Elevated plus maze test: (F) distance traveled (cm), (G) number of total arm entries, (H) entries into open arms (%), and (I) time spent in open arms (%). (J–K) Porsolt forced swim test: distance traveled (cm) for 1 min blocks of the test session on test day 1 (J) and on test day 2 (K). (L) Sucrose preference test: water intake (g) and 1% sucrose intake (g). (M) Food intake (g). Values are means ± SEM. Statistical analysis was performed by one-, two-, and three-way (repeated measures) ANOVA. * p < 0.05, SE vs. CON; † p < 0.05, LE vs. CON; # p < 0.05, SE vs. LE

Supplementary Material 3: Supplementary Table 1 Number of animals used in each experiment
Supplementary Table 2 Statistical analysis of behavioral data in socially defeated mice under post-defeat separation and cohabitation conditions
Supplementary Table 3 Statistical analysis of behavioral data in socially defeated mice under short- and long-exposure conditions of the SDS session

## Data Availability

All the data used in this study are available from the authors upon request.

## Abbreviations

CSDS: Chronic social defeat stress
SEP: Separation housing condition
COH: Cohabitation housing condition
SE: Short exposure to aggressor
LE: Long exposure to aggressor
CSDS-SI: Social interaction test in the CSDS paradigm
SUS: Susceptible
RES: Resilient
LD: Light/dark transition test
OF: Open field test
EP: Elevated plus maze test
PS: Porsolt forced swim test
TS: Tail suspension test
SP: Sucrose preference test
