# Supplementary figures and images for: Comparative analysis of procedural variations in the social defeat stress paradigm in mice: effects of post-defeat housing conditions and aggressor exposure duration
Source: Mol Brain. 2026 Jul 7;19:53. doi: 10.1186/s13041-026-01325-y (PMC13344037; doi:10.1186/s13041-026-01325-y)

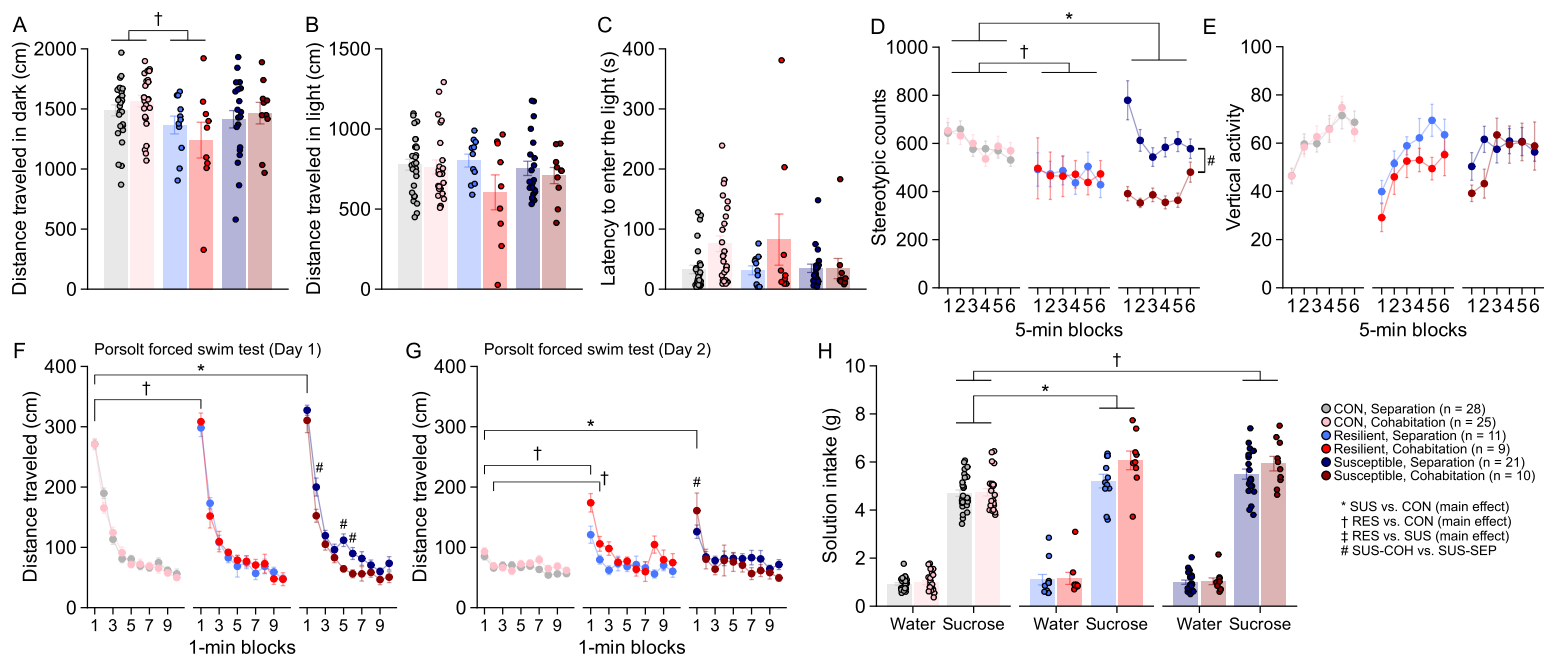

Supplement: Supplementary file 1 — Supplementary Material 1: Supplementary Figure 1 Behavioral outcomes in socially defeated mice under post-defeat separation and cohabitation conditions. (A–C) Light/dark transition test: (A, B) distance traveled in the dark and light chambers (cm) and (C) latency to enter the light chamber (s). (D–E) Open field test: (D) stereotypic counts and (E) vertical activity. (F–G) Porsolt forced swim test: distance traveled (cm) for 1 min blocks of the test session on test day 1 (F) and on test day 2 (G). (H) Sucrose preference test: water intake (g) and 1% sucrose intake (g). Values are means ± SEM. Statistical analysis was performed by one-, two-, and three-way (repeated measures) ANOVA. * p < 0.05, SUS vs. CON; † p < 0.05, RES vs. CON [file 13041_2026_1325_MOESM1_ESM.pdf]

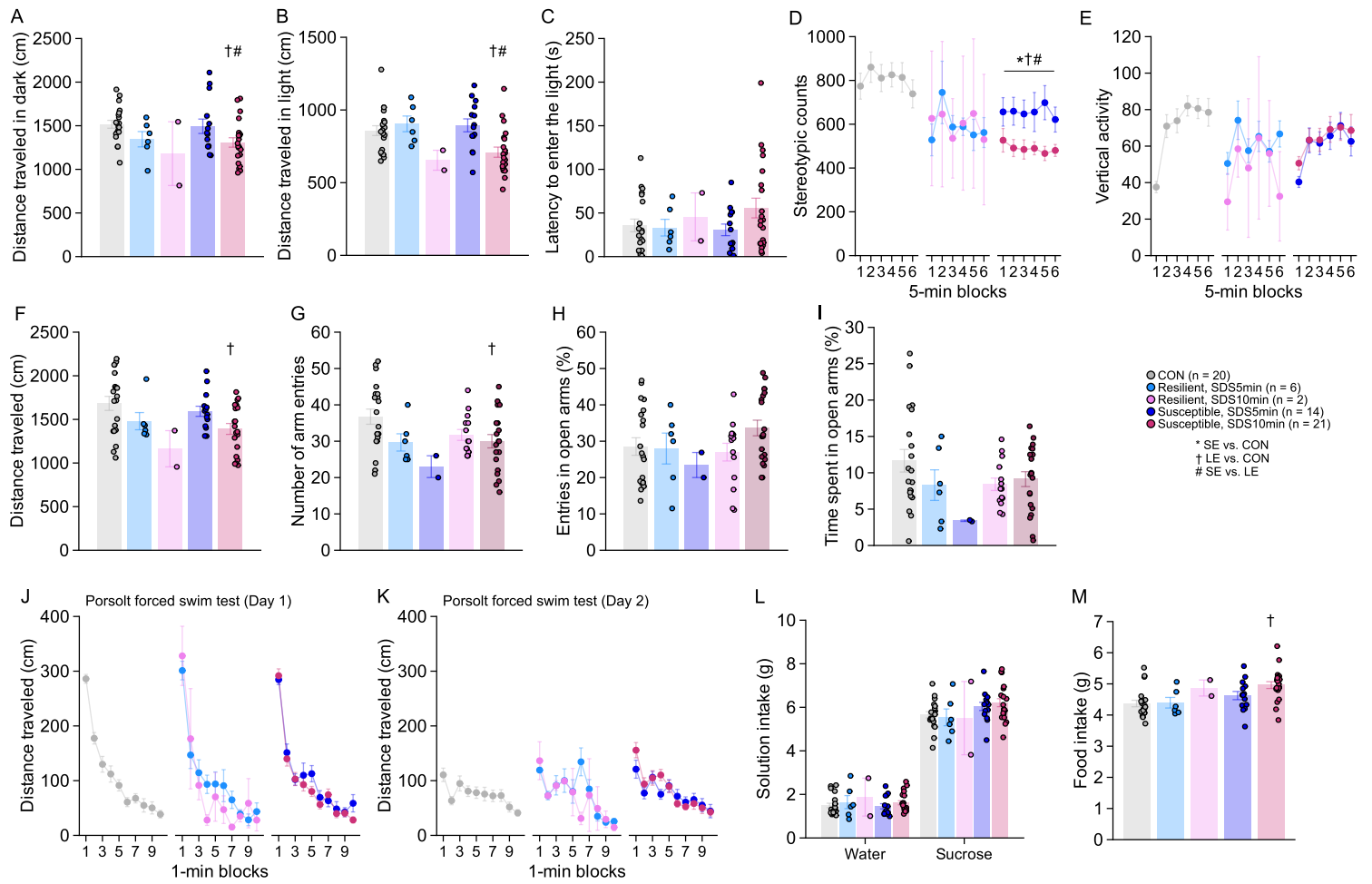

Supplement: Supplementary file 2 — Supplementary Material 2: Supplementary Figure 2 Behavioral outcomes in socially defeated mice under short- and long-exposure conditions of the SDS session. (A–C) Light/dark transition test: (A, B) distance traveled in the dark and light chambers (cm) and (C) latency to enter the light chamber (s). (D–E) Open field test: (D) stereotypic counts and (E) vertical activity. (F–I) Elevated plus maze test: (F) distance traveled (cm), (G) number of total arm entries, (H) entries into open arms (%), and (I) time spent in open arms (%). (J–K) Porsolt forced swim test: distance traveled (cm) for 1 min blocks of the test session on test day 1 (J) and on test day 2 (K). (L) Sucrose preference test: water intake (g) and 1% sucrose intake (g). (M) Food intake (g). Values are means ± SEM. Statistical analysis was performed by one-, two-, and three-way (repeated measures) ANOVA. * p < 0.05, SE vs. CON; † p < 0.05, LE vs. CON; # p < 0.05, SE vs. LE [file 13041_2026_1325_MOESM2_ESM.pdf]
